# Drug-Related Halitosis: A Narrative Review

**DOI:** 10.1016/j.identj.2025.109332

**Published:** 2026-01-09

**Authors:** Mina Iranitalab, Aviv Ouanounou

**Affiliations:** Faculty of Dentistry, University of Toronto, Toronto, Canada

**Keywords:** Halitosis, Medications, Side effects

## Abstract

**Introduction:**

This article provides a current narrative review of the medications that may cause halitosis as a side effect. Halitosis is frequently associated with important social, psychological, and emotional aspects of life; therefore, it is crucial for health care providers to be able to diagnose and manage it effectively.

**Methods:**

A literature review was conducted using the PubMed and EMBASE/OVID databases between January 2015 and December 2024 to find the latest relevant articles, focusing on systematic reviews and literature published between 2020 and 2025.

**Results:**

Medications can lead to halitosis (bad breath) either intra- or extra-orally. Research has identified several medications that may cause extra-oral halitosis as a side effect. These include ranitidine, cysteamine, certain antifungals, peppermint oil, aspirin and other NSAIDs, PX-12, silybin, disulfiram, suplatast tosilate, dimethyl sulfoxide, levocarnitine, nitrates and nitrites, paraldehyde, chloral hydrate, and iodine-containing medications. Intra-oral halitosis is mostly related to medications that cause xerostomia and MRONJ as side effects.

**Discussion:**

Multiple groups of medications can cause intra- or extra-oral halitosis. prior knowledge about these medications and their underlying mechanisms that will lead to halitosis will enable clinicians to diagnose and manage this condition more effectively. It is also wise for clinicians to consider recreational drugs like crack, cocaine, and smokeless tobacco when looking for the underlying reason for halitosis.

**Summary:**

Multiple groups of medications can act as an underlying cause for halitosis. However, more research is needed to monitor halitosis as an independent side effect and to investigate the mechanism by which each medication causes halitosis.

## Introduction

**Fig. 1:**
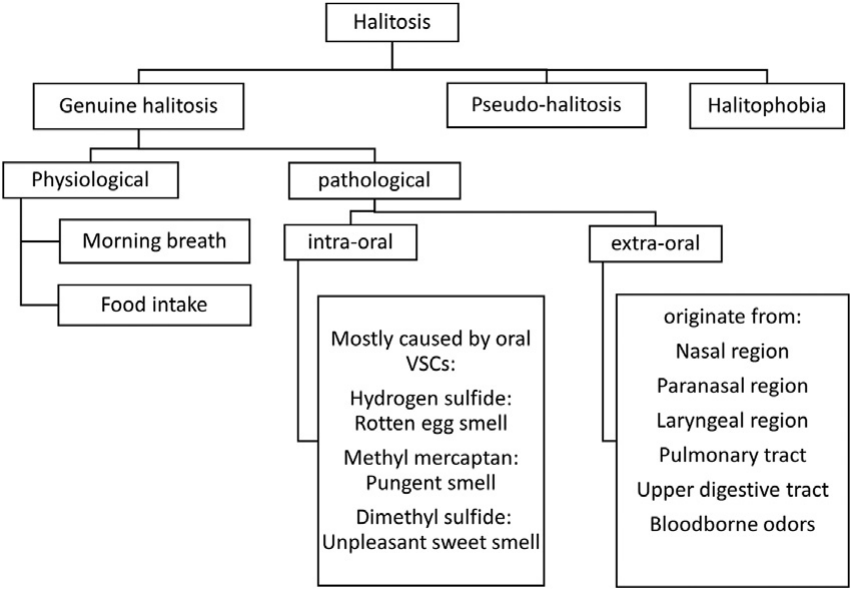
Halitosis classification.^1^^,^^10^

Halitosis is defined as a condition in which an unpleasant odour is exhaled from the oral cavity and upper airway.^1^^,^^2^ Halitosis is a prevalent condition in the population, and based on the various studies, there is a variation in the prevalence of halitosis from approximately 2.4% to 78%, and according to a systematic review, this prevalence would be 31.8%.^1^ Halitosis can be classified as genuine halitosis, pseudo-halitosis, and halitophobia. Genuine halitosis will then be subclassified as physiological and pathological halitosis. Genuine halitosis is the true halitosis and can be felt by others, while pseudo-halitosis refers to the belief of having halitosis despite the absence of any malodour. In halitophobia, patients will continue to complain about halitosis even after receiving treatment for either true or pseudo-halitosis.^2^^,^^3^ Pathological halitosis can be intra-oral or extra-oral.^4^ Please see Figure 1. Intra-oral halitosis originates from oral tissue conditions like periodontal diseases and tongue coating.^5^ Bacteria causing gingivitis and periodontitis generate volatile sulfur compounds. These are the gram-negative anaerobic bacteria in oral flora that are responsible for oral malodour by converting sulphur-containing amino acids of the oral secretions and food debris to VSCs.^5^ Lee et al.^6^ investigated the VSCs level and halitosis in patients with gingivitis and periodontitis and reported a close and strong relationship between periodontal disease and halitosis. Hydrogen sulfide (H_2_S), methyl mercaptan (CH_3_SH), and dimethyl sulfide (CH_3_SCH_3_) are the majority of VSCs that are responsible for oral malodour.^7^ Saliva plays a crucial role in preventing oral malodour. Individuals with xerostomia have increased plaque formation, and the gut flora shifts from gram-positive to gram-negative. High-viscosity saliva contains more mucus than serous saliva and contain more salivary proteins like mucins. Oral microbial species use sulphur-containing amino acids in saliva as a source of VSC and there is reduced cleaning activity associate with high viscosity saliva. As a result, these individuals are substantially more likely to develop malodour.^5^ Mucosal wounds, ulcers, and infections are among the less common causes of the halitosis^1^ and often arises as complications of Medication-Related Osteonecrosis of the Jaw. Extra-oral halitosis originates from the nasal, paranasal, and laryngeal regions, the pulmonary or upper digestive tract, and bloodborne odours.^4^^,^^5^ Approximately 3% of extraoral halitosis cases are originated on the tonsils, due to caseum accumulation on their crypts. Caseums have a morphology similar to the dental biofilm (desquamated epithelial cells, keratin and food debris) colonised by anaerobic bacteria that produces VSCs, very similar to the tongue coating microbiota which can explain the mechanism leads to malodour. Bacterial sinusitis is the main cause of malodour exhaled though nasal cavity. Bacteria involved in mucus production are capable of directly producing VSCs.^1^ Multiple systemic conditions like liver pathology, pre-kidney transplantations, renal pathology, viral hepatitis B infection, and food or medication products can cause bloodborne factors that will lead to extra-oral halitosis.^4^^,^^5^ Bloodborne halitosis is mainly the result of dimethyl sulfide circulating in the blood and exhaled during the gaseous exchange in the lungs.^1^

Halitosis is frequently associated with important social, psychological, and emotional aspects of life, and sometimes it can be considered an obstacle to social interactions. It can lead to isolation, low self-esteem, anxiety, and an overall feeling of embarrassment. All these will eventually lead to unstable mental health, poor social interaction, impediments in reaching academic goals, and an overall decreased quality of life, especially in the younger population with an active social life.^8^ As mentioned in some previous papers, halitosis can be a side effect of medications^9^^,^^10^ and prior knowledge about them will help clinicians to diagnose and manage the underlying cause of halitosis more effectively. This article will provide a narrative review of the most recent literature about medications that may cause halitosis.

## Methods

The present study was designed as a narrative review and reported following the guidelines published by Green et al.^11^ (Appendix) and Booth et al.^12^ A thematic analysis was employed through the literature review process to identify the answer to the following questions: (1) What medications can induce halitosis? In addition (2) What mechanism is leading to this side effect?

### Searching strategy

This narrative review was conducted by searching the PubMed and EMBASE/OVID databases for literature published between January 2015 and December 2024, using predefined research keywords. Grey literature was also explored through Google Scholar. The primary search identified similar studies, including those by Mortazavi et al. and Torsten et al., which informed the direction of a secondary search. The secondary search was carried out using the PubMed, EMBASE/OVID, and Google Scholar databases focusing on literature published between January 2020 and December 2024. The keywords combination used in both the primary and secondary searches is detailed in Table 1.

**Table 1:** Primary and secondary search keywords.

Databases	Search keywords
Primary search	
PubMed & EMBASE/OVID	((“drug” OR “medication” OR “medication-related”) AND (“toxicity” OR “toxicities” OR “adverse events” OR “adverse effects” OR “adverse reactions” OR “side effects” OR “side reactions”) AND (“halitosis” OR “oral malodour” OR “oral malodour” OR “bad breath” OR “foul breath” OR “fetid breath”))
Google Scholar	((“drug” OR “medication” OR “medication-related”) AND (“toxicity” OR “toxicities” OR “adverse events” OR “adverse effects” OR “adverse reactions” OR “side effects” OR “side reactions”) AND (“halitosis” OR “oral malodour” OR “oral malodour” OR “bad breath” OR “foul breath” OR “fetid breath”))
Secondary search	
PubMed & EMBASE/OVID	-((“drug” OR “medication” OR “medication-related”) AND (“gastrointestinal disorders” OR “GERD” OR “xerostomia” OR “dry mouth” OR “MRONJ” OR “medication related osteonecrosis of jaw”)) -((“Ranitdine” OR “Cysteamine” OR “Oxybutynin” OR “Glycopyrrolate” OR “Tricyclic antidepressants” OR “Antidepressants” OR “Corticosteroids” OR “NSAIDS” OR “PX12” OR “Silybin”) AND (“adverse events” OR “adverse effects” OR “adverse reactions” OR “side effects” OR “side reactions” OR (“halitosis” OR “oral malodour”))
Google Scholar	-((“drug” OR “medication” OR “medication-related”) AND (“gastrointestinal disorders” OR “GERD” OR “xerostomia” OR “dry mouth” OR “MRONJ” OR “medication related osteonecrosis of jaw”)) -((“Ranitdine” OR “Cysteamine” OR “Oxybutynin” OR “Glycopyrrolate” OR “Tricyclic antidepressants” OR “Antidepressants” OR “Corticosteroids” OR “NSAIDS” OR “PX12” OR “Silybin”) AND (“adverse events” OR “adverse effects” OR “adverse reactions” OR “side effects” OR “side reactions” OR “halitosis” OR “oral malodour”))

### Eligibility criteria

All articles retrieved through the defined search strategy will be considered eligible for inclusion, with priority given to those published between 2021 and 2025 and those presented in a systematic review format. Articles has been excluded if they meet any of the following criteria: (1) the full text is not accessible online, (2) the full text is published in a language other than English, or (3) the data presented are outdated and superseded by more recent findings identified during the literature search.

### Screening and data selection

**Figure 2:**
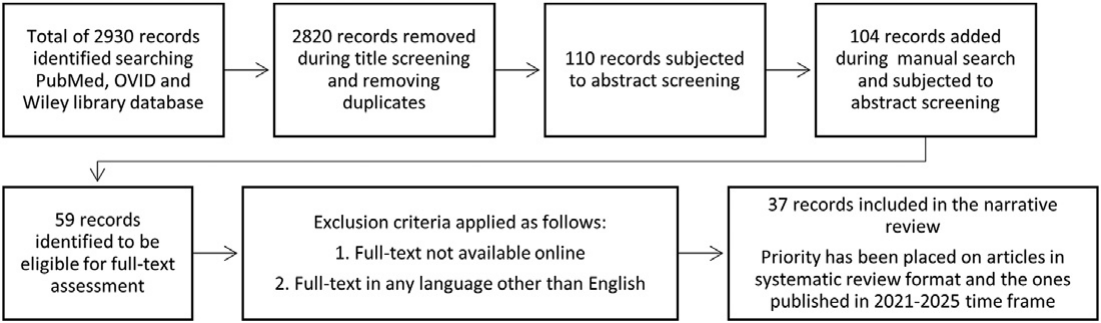
Article selection strategy.

Articles subjected to full text screening has been screened precisely and the data that could be consider helpful for clinicians for the diagnosis and treatment of halitosis was collcted, categorised and reported in 2 major group of intra- and extra- oral causations. The summary of the data selection has been shown in the Figure 2. The priority was given to the articles published between 2021 and 2025 and those presented in a systematic review format. The detail of study designs that support the data for each drug/ drug class is summarised in Table 2. One author (MI) independently carried out the search, data collection, and assembly, while the final data analysis, interpretation, and approval were conducted collaboratively by all authors.

**Table 2:** Study design of each selected study

Drug/Drug class (number of records)	References	Study design
Ranitidine (2)	Mortazavi et al.^9^ / Bodke et al.^13^	Syestematic review/ Review article
Cysteamine (6)	Joseph et al.^14^/ Klank et al.^15^/ Berends et al.^16^/ Ariceta et al.^17^/ Van Stein et al.^18^/ Bjerre et al.^19^	Clinical perspectives / Retrospective clinical trial/ Randomised, investigator-blinded, three-way cross-over trial/ Observational, retrospective, multicentre trial/ Retrospective clinical trial/ Non intervention retrospective study
Antifungals (1)	Yang et al.^20^	Systematic review
Anticholinergics and Antidepressants (3)	Lavrador et al.^21^/ Harel ^22^/ Oliva et al.^23^	Systematic review/ Master thesis/ Systematic review and meta analysis
Peppermint oil (4)	Mosaffa-Jahromi et al.^24^/ Giang et al.^25^/ Zeraattalab-Motlagh et al.^26^/ Weerts et al.^27^	Randomised control trial/ Systematic review/ Systematic review/ Randomised, double-blind trial
Xerostomia (2)	Hale et al.^28^/ *Bakhtiari et al.*^29^	N.A / A review study
GI (1)	Viana et al.^30^	Review
Tumor growth inhibitors (2)	Soleimani et al.^31^/ Ramanathan et al.^32^	An updated review/ Clinical trial
MRONJ (2)	Choi et al.^33^/ Mager et al.^34^	Clinical trial/ N.A
Disulfiram (1)	Lanz et al.^35^	Narrative review
Chloral hydrate (1)	*Izidoro et al.*^36^	Review
Suplatast tosilate (1)	Murata et al.^37^	Case report and review of the literature
Dimethylsulphoxide (2)	Harvey-Woodworth et al.^38^/ Bitcon et al.^39^	Refereed Paper/ Contemporary review
Phenothiazine (4)	Lu et al.^40^/ Alves et al.^41^/ Rubilar-Huenchuman et al.^42^/ Rakasevic et al.^43^	Review/ Overview/ Scoping review/ Prospective clinical study
Carnitine (2)	*Goin-Kochel et al.*^44^*/ Shimizu et al.*^45^	Pilot study/ Letter
Crack, Cocaine and tobacco (3)	*Muniz et al.*^46^*/ Trimarchi et al.*^47^*/ Sagiroglu et al.*^48^	Cross sectional study/ Retrospective trial/ Descriptive study

## Discussion

Medications can cause halitosis with both intra- and extra-oral origins.^3^

### Extra-oral halitosis

Extra-oral halitosis can be bloodborne or non-bloodborne. Bloodborne halitosis is caused by VSCs that are produced in distant sites and are absorbed into the bloodstream and circulated to the lungs where finally are exhaled. Non-bloodborne halitosis originates mainly from the superior and inferior respiratory tract, with a smaller contribution from gastric sources.^1^

*Ranitidine* is a histamine H_2_-receptor antagonist that will selectively block H_2_-receptors.^49^ It will decrease both the volume and concentration of the gastric acid.^13^ Mortazavi et al.^9^ in a systematic review, mentioned that ranitidine would cause extra-oral halitosis in 1 out of 110 patients if taken 150 mg daily, however; Bodke et al in an assessment and monitoring of adverse effect of ranitidine through a pharmacovigilance study, did not mention halitosis as a side effect for ranitidine and there is no complaint of oral malodour during interviews among their patients who took ranitidine for 15 to 120 days.^13^

*Cysteamine,* is used to treat cystinosis or Huntington’s disease and is known to cause extra-oral halitosis.^9^ It is a sulfur-containing compound. Treatment with cysteamine will cause VSC formation.^50^ Additionally, cysteamine is ulcerogenic and will induce GI symptoms via several mechanisms;^14^ Therefore it will contribute to extra-oral halitosis. Cysteamine doses of 15 mg/Kg and higher, or 0.9 g/m^2^ to 1.2 g/m^2^, have been shown to cause halitosis in up to 100% of the patients.^9^ Three different formulations of oral cysteamine bitartrate are available: immediate-release, delayed-release enteric-coated and the last one in which cysteamine is encapsulated by a pharmacist for enteric release (EC-cysteamine). Klank et al.^15^ concluded that 88.2% of patients taking IR-cysteamine complaint about gastrointestinal side effects like nausea, vomiting, abdominal pain, and sulfurous body odour or halitosis while with EC-cysteamine there were no reports of nausea and vomiting and 66.7 % of patients had noticed sulfurous body odour or halitosis with less discomfort comparing to other group. In another study, by Berends et al. the comparison between 3 types of cysteamine bitartrate demonstrated that 2 out of 9 subjects reported mouth odour change after taking Cystagon (immediate-release) and 1 out of 9 reported it after PO-001 (a new investigational sustained-release cysteamine) administration while there was no report of halitosis related to Procysbi, an enteric- coated cysteamine bitartrate formulation.^16^ The results of 3 other studies also indicated that a switch from IR-cysteamine bitartrate to EC-cysteamine bitartrate will improve halitosis in patients. 17, 18, 19 This improvement did not seem to be dose-related as the mean daily prescribed dose is decreased while switching from IR-cysteamine bitartrate to DR-cysteamine bitartrate in one study^19^ but an increase in dosage is mentioned in the other study.^18^

*Antifungals* are a group of medications that have halitosis as their side effect.^9^ Gastrointestinal disorders are among the etiological factors that can lead to extra-oral halitosis.^5^^,^^51^ Yang et al., in a systematic review, indicated that gastrointestinal disorders are among the side effects of nine commonly prescribed antifungals: Liposomal Amphotericin B (LAmB), Anidulafungin, Caspofungin, Fluconazole, Isavuconazole, Itraconazole, Micafungin, Posaconazole, and Voriconazole.^20^ More investigation is needed to indicate whether halitosis is a considerable side effect of antifungals and whether it is dose-related.

*Peppermint oil* is prescribed for irritable bowel syndrome treatment. It is mentioned to be associated with halitosis in 13.16% of patients by Mosaffa-Jahromi et al.^24^ in a randomised control trial. However, more recent clinical trials and systemic reviews do not support halitosis as an independent side effect although it is probable that they did not monitor it. However, they do mention several other gastrointestinal side effects such as heartburn/GERD symptoms, nausea, and belching with/ without minty taste that can be halitosis indicators.^25^, ^26^, ^27^

In summary, there are 2 major causes for bowel diseases to cause halitosis: first is an increase in the number of bacteria that reduce sulfide compounds, resulting in higher concentrations of HS and second are medications like budesonide that will cause halitosis by reducing salivary flow.^30^

*Aspirin* and other *NSAIDs* are major groups of medications that are associated with heartburn/GERD symptoms, and among the extra-oesophageal manifestations of GERD, halitosis is notable.^30^ However, there is a lack of literature to assess this probable association.

*PX-12* and *Silybin* are tumor growth inhibitors associated with halitosis.^9^^,^^31^ Dosage of 9 to 600 mg/m of PX-12 has caused halitosis in 5.5% to 100% of patients,^9^ and dosage of 2.5 to 20 mg/d of Silybin administered for a month has resulted in halitosis in 15% of the trial population.^9^^,^^31^ Both medications are also related to other gastrointestinal side effects.^31^^,^^32^

*Disulfiram* is a medication that was previously known only as a treatment for alcohol dependence. However, currently, it is also considered a treatment for inflammation, infection, cancer, and neurologic disorders.^35^ Disulfiram converts to an active metabolite, diethyldithiocarbamate (DDTC), in the stomach. In the blood, it changes to diethyldithiocarbamic acid (DDC), which is degraded to form diethylamine and carbon disulfide. Carbon disulfide can be transported from the blood into alveolar air.^10^ As a result carbon disulfide can be detected both in the blood and breath after the disulfiram intake. It could be identified in the breath after 72 hours of disulfiram intake and is an odourous volatile that can induce halitosis.^10^^,^^35^

*Suplatast tosilate* was recognised to cause halitosis in a 33-year-old Japanese woman. This was reported in a case report study by T Murata et al. It was prescribed for asthma treatment for 100mg after each meal. This medication contains the (CH3)2S structure which can enter the blood during its metabolisation and finally exhaled from the lungs and lead to halitosis. However, it seems that the amount of exhaled (CH3)2S does not exceed the social tolerance of oral malodour in most cases.^37^

*Dimethylsulphoxide (DMSO)* is a vehicle for several medications, and it is the only FDA- and Health Canada-approved intravesical agent. However, the data is conflicting on its anti-inflammatory and muscle relaxant effects. Treatment with DMSO will cause halitosis due to its metabolisation to dimethyl sulphone (DMSO2) and DMS. DMS is a volatile organosulphur compound with an exceptionally low odour detection threshold. Bacterial species known to synthesise DMS have been demonstrated in the human GI tract. DMS would not break down in the GI tract and will pass straight to the bloodstream instead. Free DMS is a neutral molecule and is unreactive with proteins, hence, it would be stable in the blood. Finally, DMS will be excreted by renal and pulmonary pathways. This may lead to a “garlic breath” odour due to DMSO treatment.^38^^,^^39^

*Levocarnitine* is a carnitine supplement that can be beneficial for multiple causes of carnitine deficiency and genetic conditions such as Rett syndrome. Goin-Kochel et al reported that administration of oral suspension or tablets of levocarnitine in 3 divided doses, starting at 200 mg/kg/d and increasing to 400 mg/kg/d, with a maximum daily dose of 6 g would be beneficial in children with autism spectrum disorder. There are two major side effects associated with this high-dose administration: fishy body/breath odour, and diarrhoea. This malodour can even lead to mid-treatment discontinuation by patients.^44^ This fishy body/breath odour is probably due to the plasma concentration of trimethylamine as a carnitine metabolite.^10^^,^^45^

In addition to all discussed medications, *Nitrates and Nitrites, Paraldehyde, Chloral hydrate,* and *iodine-containing* medications are also mentioned as examples of medications that can cause bloodborne halitosis.^10^^,^^36^ However, the generic mode of action of Paraldehyde, Chloral hydrate, and iodine-containing medications that will result in halitosis is not described in the current literature.

### Intra-oral halitosis

The oral cavity is the main cause of halitosis in 85-90% of patients.^3^ Oral cavity is a humid environment with an ideal temperature for many microorganisms to grow and metabolise amino acids to produce VSCs. The main intra-oral factors capable of causing halitosis are tongue coating, periodontitis, bad oral hygiene, and the presence of infections, caries, and mucosal wounds.^1^ Xerostomia and MRONJ are among the aetiological factors of intra-oral halitosis that are most related to medications.

Different medications can be related to Xerostomia. Anticholinergic medications are known for causing xerostomia as a side effect by blocking the muscarinic receptors that mediate glandular secretion, including saliva (M1, M3, and M4 receptors).^21^ Therefore, halitosis can always be among their side effects. Taking 5 to 15 mg/d of oxybutynin or 40 to 175 µg/d of glycopyrrolate can cause halitosis in 15.5% and 4.5% of patients, respectively^9^; however, it is not clear that if halitosis caused by named medications is related to xerostomia. Additionally, anticholinergic side effects are common in some antidepressant medications like tricyclic antidepressants, non-adrenaline reuptake inhibitors, Paroxetine, Opipramol, and Trazodone.^22^ Oliva et al.,^23^ in a systemic review, reported dry mouth among patients who are taking Buproprion, Desvenlafaxine, Duloxetine, Escitalopram, Fluvoxamine, Levomilnacipran, Paroxetine, Reboxetine, Sertraline, Venlafaxine. Other gastrointestinal disorders such as nausea and vomiting, diarrhoea, constipation, abdominal pain, dyspepsia, anorexia, and increased appetite are among the side effects of antidepressant medications,^23^ therefore, it is not clear that the probable halitosis is intra- or extra-oral. Overall, hundreds of medications can cause xerostomia. Still, the most notable ones are those that are prescribed to treat depression, high blood pressure, anxiety, and pain, as well as antihistamines, decongestants, and muscle relaxants.^28^ Additionally, Appetite suppressants, supplements, non-steroid anti-inflammatory drugs (NSAIDs), systemic retinoids, anti-viral drugs, and medications like cytokines (interferon α, interleukin 2) can cause xerostomia to some extent.^29^ Highlighting these medications can be helpful for diagnostic purposes.

Literature is controversial about the association of Phenothiazine and halitosis. Although previous literature believes that systemic consumption of Phenothiazine will result in halitosis due to xerostomia and occasionally white and black hairy tongue,^40^ more recent articles mention Phenothiazine among the halitosis treatments. These studies mention that photodynamic therapy with Phenothiazine derivatives like Phenothiazine chloride, methylene blue, and toluidine blue, followed by red light (580-680 nm) irradiation, will decrease the number of microorganisms (e.g. Enterococcus faecalis) that will cause intra-oral halitosis.^41^^,^^42^ D. Rakasevic et al.^43^ in a prospective study concluded that phenothiazine-derivate methylene blue (concentration of 9 mg/ml) followed by diode laser (λ = 660 nm) for 30s/point will result in a statistically significant reduction in *C. albicans* and *C. krusei* compared to NYS.

MRONJ is the other aetiological factor of intra-oral halitosis. In a recent research study, Choi et al. showed the level of halitosis in MRONJ patients as an objective value. They conclude that hydrogen sulfide and methyl mercaptan concentrations significantly differ between the MRONJ group and the osteoporosis and healthy control groups. In addition, there is a significant difference in the hydrogen sulfide concentration based on whether solid cancer causes MRONJ or osteoporosis. However, the MRONJ site and the medication type and dose did not affect the VSC concentrations in the oral cavity.^33^ Antiresorptives like bisphosphonates, including Zoledronate and Pamidronate, and RANK-L inhibitors, including Denosumab, in addition to Antiangiogenic agents, including Bevacizumab, Aflibercept, Sunitinib, Cabozantinib, Sorafenib, and Dasatinib, are among the medications that are related to osteonecrosis of the jaw. Additionally, Raloxifene, Methotrexate, and Tocilizumab have demonstrated a link with MRONJ.^52^ Methotrexate can have other adverse effects that may result in halitosis, like gingivitis, anorexia, diarrhoea, and ulcers with or without GI bleeding. ^34^

Penicillamine is an essential drug for treating rheumatoid arthritis. It is also a product of penicillin antibiotics and can affect the oral cavity. Its metabolism raises the pH level, favouring the proliferation of gram-negative bacteria in the oral cavity, and the interaction between these bacteria and specific substrates will produce odourous compounds that may cause oral malodour.^10^

In addition to prescribed medications, recreational drugs like crack can lead to higher assurance of self-report halitosis, and this can be due to reduced salivary flow, greater number of decayed teeth^46^, and/or other intra-oral consequences. Halitosis is mentioned among the most common symptoms of patients with a history of cocaine use who are currently present with palatal perforation.^47^ It is also noteworthy that halitosis was 9.4 times more common among smokeless tobacco users than among non-tobacco users. This is assumed to be due to the stimulation of *S. mutans* colonisation and the caries rate increase.^48^

### Clinical application

Among the various classifications of halitosis, genuine halitosis is the type that dentists, as healthcare providers, should focus on treating. The underlying causes of halitosis are categorised into intra-oral and extra-oral origins, with intra-oral factors being more common. Therefore, it is clinically prudent to begin the diagnostic process within the oral cavity. Key intra-oral contributors include poor oral hygiene, tongue coating, periodontal disease, dental caries, xerostomia, gingivitis, salivary gland pathosis, oral ulcers, oral cancers, fixed orthodontic appliances, low salivary flow rate, high salivary viscosity, and faulty dentures.^5^ These conditions should be thoroughly evaluated, prevented and managed by dentists before considering extra-oral causes or referrals.

Box 1 **Steps for management halitosis summary**

- Update patient’s medical and medication history
- Manage and roll out intra-oral contributing causes:
  • Improve oral hygiene: improve brushing and flossing techniques, using mouth washes, reduce tongues coating.
  • Manage gingivitis, periodontitis and dental caries.
  • Control xerostomia and its underlying cause: more frequent hydration, using saliva substitutes.
  • Manage other intra-oral factors: oral ulcers, faulty dentures.
- Consider extra-oral contributing causes and appropriate referrals:
  • ENT region: adenoid hypertrophy, allergic rhinitis.
  • GI tract: peptic ulcer.
  • Blood borne factors: liver pathology, pre-kidney transplantation, renal pathology, viral B hepatitis, food products, medications.

Alt-text: Unlabelled box dummy alt text

The treatment of halitosis should begin with improving oral hygiene practices, including regular brushing, flossing, and the use of mouthwashes when appropriate. Mouthwashes can be categorised as either cosmetic or therapeutic. Cosmetic mouthwashes lack bactericidal or bacteriostatic properties and are primarily used to temporarily mask symptoms such as bad breath. In contrast, therapeutic mouthwashes contain active antimicrobial ingredients such as cetylpyridinium chloride, chlorhexidine, fluoride, or hydrogen peroxide that help reduce oral bacterial load. These antimicrobial agents may serve as adjuncts in halitosis management by either masking malodour or addressing its underlying bacterial causes, although clinical evidence remains limited.^53^ Implementing these approaches can contribute to a reduction in dental caries and periodontal diseases.

**Table 3:** Summary of medication contributing to halitosis.

Extra-oral halitosis	Intra-oral halitosis	
	Xerostomia	Anticholinergics
		Antidepressants
		Antihistamines
		Antihypertensives
Ranitidine		Antipsychotics
Cysteamine		Anxiolytics
Antifungals		Antivirals
Peppermint oil		Muscle relaxants
Aspirin and NSAIDs		Decongestants
PX-12		Appetite suppressants,
Silybin		NSAIDs
Disulfiram		Systemic retinoids
Suplatast tosilate		Medications like cytokines
Dimethylsulphoxide	MRONJ	Antiresorptives
Levocarnitine		RANK-L inhibitors
Nitrates and Nitrites		Antiangiogenic agents
Paraldehyde		Raloxifene
Chloral hydrate		Methotrexate
Iodine-containing medications		Tocilizumab
	Other	Penicillamine
		Recreational drugs: crack, cocaine, smokeless tobacco

As previously discussed, saliva plays an important role in maintaining oral health and function, including speech, mastication, swallowing, and the prevention of dental caries. A deficiency in saliva can be a significant contributing factor to halitosis. Xerostomia, or dry mouth, may result from various underlying causes such as radiation therapy to the head and neck region; systemic diseases like Sjögren’s syndrome, rheumatoid arthritis, and diabetes mellitus; or salivary gland disorders due to trauma or oncologic conditions. However, the most common cause of hyposalivation is medication-induced, particularly among older adults who often take multiple medications to manage chronic systemic diseases. An effective diagnostic approach for xerostomia includes measuring both unstimulated and stimulated salivary flow rates. While the main cause of xerostomia may not always be manageable, the condition can often be controlled through strategies such as encouraging more frequent hydration and recommending the use of saliva substitutes.^54^

After evaluating and addressing intra-oral factors, if halitosis persists, it is advisable to consider extra-oral causes such as medication-induced halitosis and proceed with appropriate referrals when necessary.

### Limitations

Overall, more research is necessary to assess and monitor halitosis as a side effect of drug consumption. Only a few assessments and monitoring have been conducted that follow halitosis as an independent side effect, and the ones that are available did not clearly mention the extra- or intra-oral origin. More research is required to clarify available data and help clinicians diagnose and manage halitosis more accurately.

## Summary

Halitosis can be due to consuming some specific medications, and its origin can be intra-oral or extra-oral. Research showed that Ranitidine, Cysteamine, some Antifungals, Peppermint oil, Aspirin and NSAIDs, PX-12, Silybin, Disulfiram, Suplatast tosilate, Dimethylsulphoxide, Levocarnitine, Nitrates and Nitrites, Paraldehyde, Chloral hydrate, and iodine-containing medications are among the medications that can be considered to cause extra-oral halitosis as their side effect. Xerostomia and MRONJ are common side effects of medications that can lead to intra-oral halitosis; therefore, prior knowledge of medications that are more likely to cause these conditions can help clinicians diagnose and manage the underlying causes of halitosis more effectively. It is also helpful to have recreational drugs like crack, cocaine, and smokeless tobacco and their oral side effects in mind while looking for the underlying reason for halitosis. Medications that contribute to intra- or extra-oral halitosis are summarized in Table 3.

## Author contributions

Conception and design: Aviv Ouanounou. Administrative support: A Ouanounou. Provision of study materials or patients: Mina Iranitalab. Collection and assembly of data: Mina Iranitalab. Data analysis and interpretation: Aviv Ouanounou and Mina Iranitalab. Manuscript writing: Aviv Ouanounou and Mina Iranitalab. Final approval of manuscript: Aviv Ouanounou and Mina Iranitalab.

## Funding

This research did not receive any specific grant from funding agencies in the public, commercial, or not-for-profit sectors.

## Conflict of interest

None disclosed.
